# Value of contrast-enhanced CT texture analysis in predicting IDH mutation status of intrahepatic cholangiocarcinoma

**DOI:** 10.1038/s41598-021-86497-4

**Published:** 2021-03-25

**Authors:** Yong Zhu, Yingfan Mao, Jun Chen, Yudong Qiu, Yue Guan, Zhongqiu Wang, Jian He

**Affiliations:** 1 Department of Radiology, Affiliated Hospital of Nanjing University of Chinese Medicine, Nanjing, 210029 Jiangsu Province China; 2 Department of Radiology, Nanjing Drum Tower Hospital, The Affiliated Hospital of Nanjing University Medical School, No. 321 Zhongshan Road, Nanjing, 210008 Jiangsu Province China; 3 Department of Pathology, Nanjing Drum Tower Hospital, The Affiliated Hospital of Nanjing University Medical School, No. 321 Zhongshan Road, Nanjing, 210008 Jiangsu Province China; 4 Department of Hepatopancreatobiliary Surgery, Nanjing Drum Tower Hospital, The Affiliated Hospital of Nanjing University Medical School, No. 321 Zhongshan Road, Nanjing, 210008 Jiangsu Province China; 5 School of Biomedical Engineering, Shanghai Jiao Tong University, No. 1954 Huashan Road, Shanghai, 200000 China

**Keywords:** Cancer, Risk factors, Mathematics and computing

## Abstract

To explore the value of contrast-enhanced CT texture analysis in predicting isocitrate dehydrogenase (*IDH*) mutation status of intrahepatic cholangiocarcinomas (ICCs). Institutional review board approved this study. Contrast-enhanced CT images of 138 ICC patients (21 with *IDH* mutation and 117 without *IDH* mutation) were retrospectively reviewed. Texture analysis was performed for each lesion and compared between ICCs with and without *IDH* mutation. All textural features in each phase and combinations of textural features (p < 0.05) by Mann–Whitney U tests were separately used to train multiple support vector machine (SVM) classifiers. The classification generalizability and performance were evaluated using a tenfold cross-validation scheme. Among plain, arterial phase (AP), portal venous phase (VP), equilibrium phase (EP) and Sig classifiers, VP classifier showed the highest accuracy of 0.863 (sensitivity, 0.727; specificity, 0.885), with a mean area under the receiver operating characteristic curve of 0.813 in predicting *IDH* mutation in validation cohort. Texture features of CT images in portal venous phase could predict *IDH* mutation status of ICCs with SVM classifier preoperatively.

## Introduction

Intrahepatic cholangiocarcinoma (ICC), which accounts for 5–10% of primary liver cancers, is the second most frequent primary hepatic malignancy in adults after hepatocellular carcinoma^1,2^. Isocitrate dehydrogenase (IDH), as the key enzyme in the tricarboxylic acid cycle, is the center of the material and energy metabolism^3^. To date, *IDH1/2* represented the most frequently mutated metabolic enzyme genes in human cancers^4,5^. *IDH1/2* mutations, which occurred frequently in ICCs (10–28%)^6,7^, play an important role in carcinogenesis and development of ICCs, and hold great prognostic significance^7–10^. Moreover, recent years have witnessed the identification of novel therapeutic targets in ICC including fibroblast growth factor receptor fusions and *IDH1/2* mutations^11^. There has been a consistent increase in the number of available ICC models investigating: (1) carcinogenesis processes from initiation to progression; and (2) tools for personalized therapy and innovative therapeutic approaches, including chemotherapy and immune/targeted therapies^12^. However, the establishment of preclinical models to accurately assess *IDH* mutations in ICC has become a new challenge.

Imaging modality, such as computed tomography (CT), magnetic resonance imaging (MRI), is routinely used for preoperative evaluation and treatment planning in ICC patients. Image-based texture analysis, which relies on computer-assisted measurements, could analyze gray-level patterns within the tissue which are imperceptible to human eyes^13^. Texture analysis has been widely used in solid tumors of head and neck, lung, kidney, pancreas and gastrointestinal tract to predict biological behavior^14^, molecular features^15–19^ and patients’ prognosis^20,21^. Nevertheless, the application of texture analysis to identify *IDH* mutation status of ICCs has never been reported.

Jakola et al.^22^ reported that by using Haralick texture parameters based on preoperative clinical fluid attenuated inversion recovery (FLAIR) sequence, the homogeneity parameter could separate *IDH* mutated low-grade gliomas from *IDH* wild tumors. Yu et al.^23^ believed that *IDH1* mutation of grade II glioma could be evaluated noninvasively by texture analysis of conventional T2-FLAIR MR images. However, application of CT image based texture analysis has never been reported in predicting *IDH* mutation status of ICCs. Previous study indicated that contrast-enhanced CT images display multiple features significantly associated with *IDH* mutation status in ICCs^24^. This finding prompts us to further excavate the correlations between CT image based texture features and *IDH* mutation status of ICCs.

Therefore, the purpose of this study was to explore the role of texture features based on multiphase contrast-enhanced CT images in predicting *IDH* mutation status of ICCs preoperatively.

## Materials and methods

The study protocol was in complies with the Declaration of Helsinki and acts in accordance to ICH GCP guidelines. Institutional review board of Nanjing Drum Tower Hospital, the affiliated hospital of Nanjing University Medical School approved this study and explicitly waived the informed consent due to its retrospective nature. It also clarified that authors had access to identifying patient information when analyzing the data.

### Patients

From January 2010 to December 2019, a total of 212 patients with a clinical diagnosis of ICC were reviewed. The inclusion criteria were: (a) with a diagnosis of ICC according to the 2010 WHO classification confirmed by pathology through exploratory laparotomy, needle biopsy or postoperative specimen; (b) with completely preoperative contrast-enhanced CT images; (c) without any local or systematic treatment history of such as percutaneous ethanol injection, radiofrequency ablation, transcatheter arterial chemoembolization, radiotherapy or chemotherapy before CT examination. The exclusion criteria were: (a) with history of malignancy or other malignant tumors; (b) with artifacts or chaotic lesions on the CT image, resulting in poor CT image quality; (c) with a failure to read images by texture analysis software because of mismatch original image parameters.

The remaining 138 patients (99 men and 39 women) served as our study cohort with a median age of 59.3 years (range 33.4–79.1). 131 patients had a solitary lesion and 7 patients had multiple lesions, with a median size of 5.3 cm (range 2.0–10.5). There were 4, 7, 84, 30, and 13 ICCs with high, high-medium, moderate, moderate-poor, and poor differentiation degree, respectively. There were 63, 20, 31 and 24 ICCs in T1, T2a, T2b, and T3 stage, respectively.

### CT examination

All patients underwent unenhanced and dynamic contrast-enhanced CT scans on a multidetector CT scanner (Lightspeed, VCT, or Discovery HD750, GE Healthcare, US). The scanning parameters were the same as detail in our team’s previous study^24^. The medium interval between CT examination and surgery was 9.2 days (range 4.7–23.3).

### CT texture analysis and support vector machine (SVM)

Plain, arterial, portal venous and equilibrium phase CT images of all patients were downloaded through a picture archiving and communication system (PACS) and uploaded into in-house software written in Python (Pyradiomics version: stable; https://github.com/Radiomics/pyradiomics). Two radiologists who were blinded to ICC *IDH* mutation status, manually drew along the margin of the tumor independently. In three patients with multiple lesions, only the largest lesion was analyzed to avoid selection bias.

According to previous studies^25–27^, the boundary of ICCs and adjacent liver parenchyma could be reliably distinguished on multiphase dynamic contrast-enhanced CT images. Most ICCs presented as irregular masses with low attenuation and incomplete rim enhancement during AP and VP, and persistent enhancement during EP. Therefore, each reader performed region of interest (ROI) delineation simultaneously in the plain, AP, VP and EP images and referred to each other phase. ROIs were manually drawn along the margin of the lesion on each axial slice (mean volume 91,309.78 mm^3^, range 1248.62–474,419.00 mm^3^), which included visible necrosis and blood vessels within the tumor, excluding adjacent liver parenchyma.

The software automatically read the CT value of each pixel within the volume of interest (VOI) and generated a set of parameters as follows: (1) the first-order features describing the distribution of pixel intensity within the VOI, including the fifth, 10th, 25th, 50th, 75th and 90th percentiles (nth percentile grey-level intensity of a cumulative histogram), entropy (the distribution of grey levels over the VOI), kurtosis (peakedness of the histogram distribution), max frequency (the peak value of a histogram), mean attenuation (mean grey-level intensity), mode (the gray level value that appears most frequently in a histogram), skew (asymmetry of the histogram distribution), and standard deviation (spread of distribution); (2) the second-order features from the grey level co-occurrence matrix (GLCM), which is a matrix with row *i* and column *j* ranging from 0 to N_g_, the number of discrete grey levels within the volumes of interest. The normalized GLCM element *p* (*i*, *j*) describes the probability of a pair of grey levels that are separated by a certain distance in a certain direction^28^. In this work, the distance between the pair was one voxel and the directions were 0°, 45°, 90°, and 135°, respectively. Texture features calculated from the GLCMs were then averaged over the four directions to eliminate any directional dependence^28,29^. Those features were contrast, correlation, energy, entropy (H) and homogeneity, which were calculated as follows:

1 $${\text{Contrast}} = \sum\limits_{i,j} {\left| {i - j} \right|^{2} } p\left( {i,i} \right)$$

2 $${\text{Correlation}} = \sum\limits_{i,j} {\frac{{\left( {i - \mu } \right)\left( {j - \mu } \right)p \left( {i,j} \right)}}{{{\upsigma }2}}}$$

3 $${\text{Energy}} = \sum\limits_{i,j} {p\left( {i,j} \right)^{2} }$$

4 $${\text{Entropy }}\left( {\text{H}} \right) = \sum\limits_{i,j} {p\left( {i,j} \right)} \;{\text{log}}_{{2}} \;p\left( {i,j} \right)$$

5 $${\text{Homogeneity}} = \sum\limits_{i,j} {\frac{1}{{1 + \left( {{\text{i}} - {\text{j}}} \right)^2}}p\left( {i,j} \right)}$$

where σ is the standard deviation of GLCM element. The measurements of each feature including thirteen first-order features and five second-order features obtained by the first radiologist were calculated for statistical analysis. The other observer repeated image analysis independently as discussed 1 month later in order to assess the intra-observer reliability for all the features.

Texture parameters were further used to build classifiers. Support vector machines (SVMs), as relatively new type of learning algorithm, were chosen as classifiers in predicting *IDH* mutation. Their remarkably robust performance with respect to sparse and noisy data is making them the system of choice in a number of applications from text categorization to protein function prediction. SVM shows good robustness and high precision, and it has been used by other study for cancer analysis^23^.

### Determination of IDH mutation status

The *IDH* mutational status was analyzed as previously study described in detail^24^. Finally, *IDH* mutation was detected in 21/138 (15.2%) patients of ICCs, including 14 cases with *IDH*1 and 7 with *IDH*2 mutation. Hence, 138 patients were divided into *IDH* mutation (+) group (n = 21, 15.2%) and *IDH* mutation (−) group (n = 117, 84.8%).

### Statistical analyses

Statistical analyses were performed with SPSS (version 22.0 for Microsoft Windows × 78, SPSS, Chicago, US). Student t test or Mann–Whitney U test were used to compare the value of each texture feature for differentiating *IDH* mutation (+) and *IDH* mutation (−) group, when appropriate. All texture features in each phase and combinations of textural features (p < 0.05) by Mann–Whitney U tests were separately used to train multiple SVM classifiers. The classification generalizability and performance were evaluated using a tenfold cross-validation scheme of randomly splitting the data into training and testing sets^30^. Receiver operating characteristic (ROC) curve and area under the ROC curve (AUC) were also used to show the overall performance of the radiomics approach. Mean sensitivity, specificity and accuracy of the classification results were calculated for each tested condition. Interobserver agreement of each CT textural features between two radiologists were assessed with intraclass correlation coefficients (0.000–0.200, poor; 0.201–0.400, fair; 0.301–0.600, moderate; 0.601–0.800, good; 0.801–1.000, excellent). A two-tailed p value less than 0.05 was considered statistically significant.

### Ethics approval and consent to participate

Institutional review board of Nanjing Drum Tower Hospital, the affiliated hospital of Nanjing University Medical School approved this study and explicitly waived the informed consent due to its retrospective nature. It also clarified that authors had access to identifying patient information when analyzing the data.

## Results

Multiple texture parameters, including entropy and standard deviation in plain CT images, 75th percentile and mode in arterial phase images, 50th percentile, 75th percentile, 90th percentile, mode and standard deviation in portal venous phase images, entropy and standard deviation in equilibrium phase images, showed certain differences between ICCs with and without *IDH* mutation (all p < 0.05, Table 1), which were further used to build Sig classifier of SVM.

**Table 1:** Mann–Whitney U test of each texture feature in multiphase contrast-enhanced CT imaging in differentiating ICC with isocitrate dehydrogenase (IDH) mutation from those without.

Texture feature	Plain	AP	VP	EP
5th percentile	0.731	0.731	0.651	0.085
10th percentile	0.651	0.229	0.731	0.254
25th percentile	0.813	0.303	0.302	0.651
50th percentile	0.731	0.731	0.041*	0.813
75th percentile	0.781	0.047*	0.035*	0.813
90th percentile	0.302	0.065	0.030*	0.813
Entropy	0.035*	0.731	0.731	0.045*
Kurtosis	0.731	0.731	0.731	0.302
Max frequency	0.302	0.302	0.731	0.302
Mean	0.731	0.731	0.085	0.731
Mode	0.355	0.027*	0.027*	0.227
Skew	0.731	0.731	0.302	0.731
Standard deviation	0.035*	0.731	0.008*	0.043*
Contrast	0.302	0.302	0.058	0.731
Correlation	0.333	0.731	0.285	0.732
Energy	0.705	0.731	0.702	0.802
Entropy (H)	0.452	0.796	0.302	0.333
Homogeneity	0.302	0.055	0.331	0.331

*AP* arterial phase, *VP* portal venous phase, *EP* equilibrium phase.

Data are p value; *p < 0.05.

**Figure 1:**
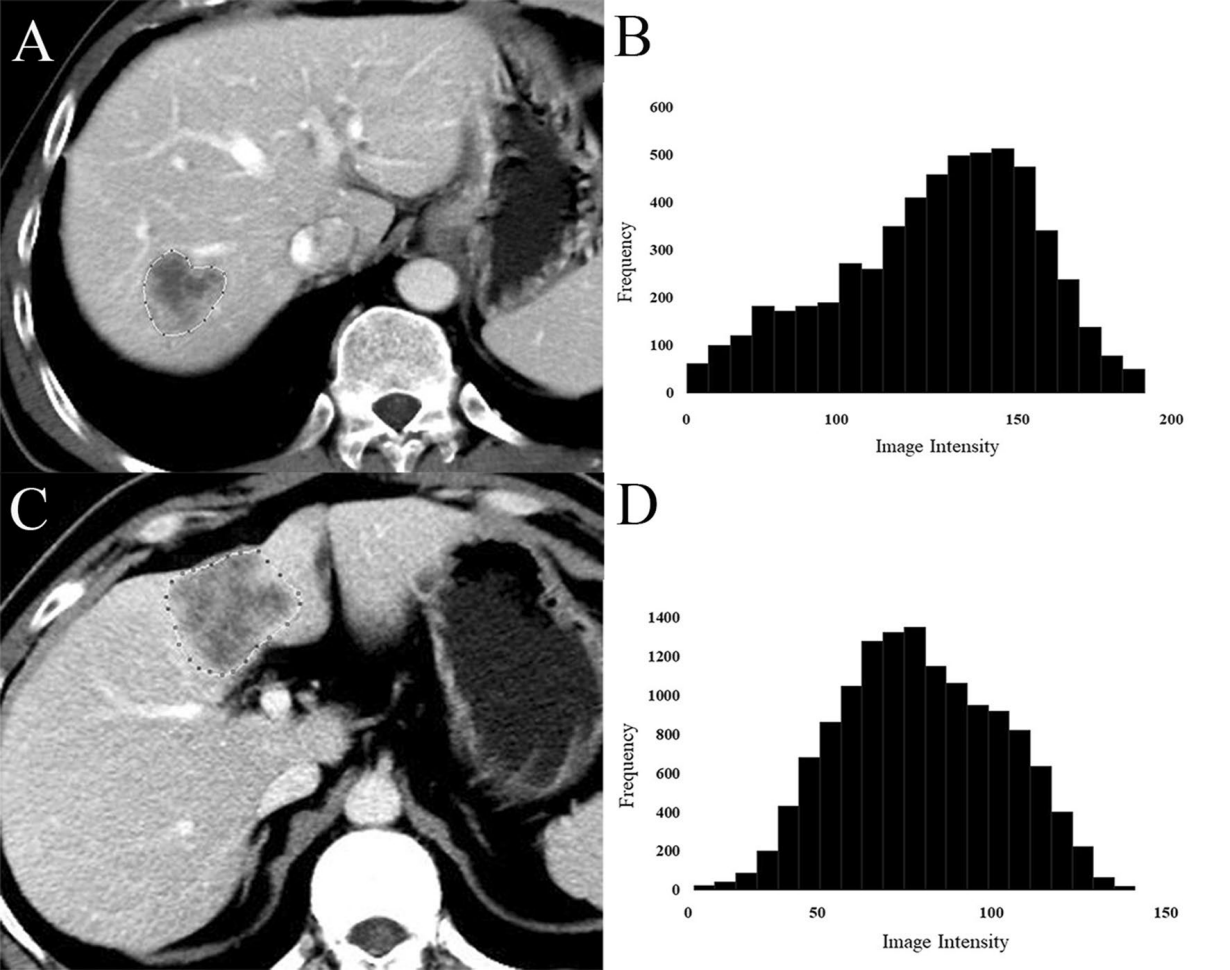
Representative cases in the portal venous phase to show texture differences between different isocitrate dehydrogenase (IDH) states. (**A**) Typical case in an IDH wild-type group; (**B**) histogram plot of the intensity values from the region of interest highlighted in (**A**). (**C**) Typical case in an IDH mutation group; (**D**) histogram plot of the intensity values from the region of interest highlighted in (**C**).

The diagnostic performance of each classifier on training cohort and tenfold cross-validation cohort are shown in Table 2. Among five classifiers, VP classifier showed the highest accuracy of 0.863 (sensitivity, 0.727; specificity, 0.885), with a mean AUC of 0.813 in predicting *IDH* mutation in validation cohort. Two representative cases are shown in Fig. 1, whose *IDH* mutation status could be correctly predicted by using VP classifier.

**Table 2:** Diagnostic performance of each classifier in training and validation cohorts.

Model	AUC^a^		Accuracy^a^		Sensitivity^a^		Specificity^a^	
	Training cohort	Validation cohort	Training cohort	Validation cohort	Training cohort	Validation cohort	Training cohort	Validation cohort
Plain classifier	0.855	0.570	0.945	0.755	0.921	0.560	0.949	0.788
AP classifier	0.920	0.657	0.905	0.793	1.000	0.517	0.890	0.839
VP classifier	0.963	0.813	0.976	0.863	1.000	0.727	0.973	0.885
EP classifier	0.769	0.544	0.974	0.766	1.000	0.660	0.970	0.784
Sig classifier	0.896	0.651	0.913	0.761	1.000	0.573	0.899	0.792

*AP* arterial phase, *VP* portal venous phase, *EP* equilibrium phase.

^a^*AUC* mean area under the curve.

Most texture features showed excellent interobserver agreement with intraclass correlation coefficients ≥ 0.80 (Table 3).

**Table 3:** Inter-observer agreement of texture parameters in multiphase contrast-enhanced CT imaging.

Feature	Plain	AP	VP	EP
5th percentile	0.770	0.882	0.894	0.865
10th percentile	0.737	0.804	0.813	0.827
25th percentile	0.771	0.916	0.920	0.923
50th percentile	0.765	0.858	0.879	0.906
75th percentile	0.749	0.835	0.866	0.880
90th percentile	0.733	0.813	0.829	0.882
Entropy	0.736	0.846	0.865	0.926
Kurtosis	0.710	0.771	0.788	0.783
Max frequency	0.694	0.954	0.896	0.882
Mean	0.853	0.856	0.882	0.877
Mode	0.744	0.888	0.918	0.916
Skew	0.802	0.874	0.928	0.708
Standard deviation	0.876	0.933	0.954	0.969
Contrast	0.737	0.839	0.876	0.706
Correlation	0.829	0.891	0.946	0.953
Energy	0.774	0.985	0.913	0.916
Entropy (H)	0.676	0.890	0.876	0.626
Homogeneity	0.797	0.926	0.915	0.915

Data are interclass correlation coefficients.

## Discussion

Our study explored the correlation between preoperative CT texture parameters and *IDH* mutation status of ICC. To the best of our knowledge, it was the first application of CT image based texture analysis in evaluating ICCs.

CT is the most commonly used imaging modality in preoperative assessment of liver tumors, which yields stable and reliable images. Texture analysis has been widely applied on plain and contrast enhanced CT images of liver^31^ for automated recognition of liver tissues, computer-assisted characterization of focal liver lesions, identification of occult malignancy, and indirect assessment of hepatic vascularity^31–37^. Texture analysis based on CT images has also been used to identify KRAS mutations of colorectal cancers^38^ and EGFR mutation status in adenocarcinoma of the lung^18,39,40^.

In this study, we extracted first- and second-order statistical features based on multiphase contrast enhanced CT imaging^41^, which not only reflects the distribution of pixel values within the ICC's volume of interest, but also describes the pattern of voxel spatial distribution. Texture features enabled the description of the variations in the surface intensity or patterns at the lesion area, including some that are indiscernible to the human eye^13^. Additionally, texture parameters in our study were derived from whole tumor volume, which might reflect tumor microstructures and heterogeneity better than texture analysis based on a single slice^35^.

In this study, different SVM classifiers were established to distinguish ICCs with *IDH* mutation from those without, including plain classifier, arterial phase classifier, portal venous phase classifier, equilibrium phase classifier and Sig classifier. SVM was chosen owning to its good robustness and high precision^42^. Yu et al.^23^ found that SVM classifier based on conventional T2-FLAIR images performed better than Adaboost in predicting *IDH1* status of grade II glioma with an AUC of 85.72%. Li et al.^43^ reported that leave-one-out cross-validation SVM based on multiple-modality MR images-based deep learning-based radiomics could predict *IDH1* status of low-grade glioma with an AUC of 95%.

We found that the portal venous phase classifier performed best in identifying ICCs with *IDH* mutation from those without, which indicated that texture features in portal venous phase provided more valuable information in assessing *IDH* status in ICCs. Previous study found that the maximum CT value of the tumor in portal venous phase could distinguish ICCs with *IDH* mutation from those without^24^.

In recent years, the advent of molecular sequencing has paved the way toward a potential new era in ICC management^11^. The most promising therapeutic options for ICC originate from targeted therapies, including *IDH* inhibitors. The identification of key oncogenic drivers in ICC has become a prerequisite for studying the applicability of immunotherapy. As topical issues, radiomics methods have already been widely adopted for the noninvasive analysis of genetic and clinical information in different medical fields. The success of radiomics is based on the idea that medical images can provide much information about the internal state, which could be related to genotype and may help in treating and understanding disease. Due to the rarity of the disease, the interdisciplinary collaboration of the ICC scientific community is essential.

This study had some limitations. First, this is a single-center retrospective study of the Asian population. Due to the low incidence of ICC, the sample size is relatively small, further analysis based on a larger sample size is required to confirm our findings. Second, CT images were obtained from different CT scanners, which might bring some potential bias. Nevertheless, a good inter-scanner agreement of CT texture analysis has been confirmed in previous study^44^. Third, texture parameters were derived from ROIs drawn manually by radiologists, since automatic segmentation of ICCs proved quite difficult due to its irregular margins. Nevertheless, most texture features in our study showed excellent interobserver agreement with intraclass correlation coefficients ≥ 0.80. Forth, the SVM classifier model might be overfited due to lack of a separate validation cohort. Nevertheless, a tenfold cross validation method was utilized to verify the performance of the classifier, avoiding over- or under-fitting and improving the generalizability of the model. Fifth, the predictive power of each single feature is not explored in this study, we are planning to investigate this issue further.

In conclusion, we confirmed that by using SVM classifier, texture parameters derived from portal venous phase CT images could predict *IDH* mutation status in ICCs preoperatively.

## Data Availability

The datasets during and/or analysed during the current study available from the corresponding author on reasonable request.

## References

[1] Khan SA, Thomas HC, Davidson BR, Taylor-Robinson SD (2005). Cholangiocarcinoma. Lancet.

[2] Liver Cancer Study Group of Japan (1990). Primary liver cancer in Japan. Clinicopathologic features and results of surgical treatment. Ann. Surg..

[3] Prensner JR, Chinnaiyan AM (2011). Metabolism unhinged: IDH mutations in cancer. Nat. Med..

[4] Yan H, Parsons DW, Jin G, McLendon R, Rasheed BA, Yuan W, Kos I, Batinic-Haberle I, Jones S, Riggins GJ, Friedman H, Friedman A, Reardon D, Herndon J, Kinzler KW, Velculescu VE, Vogelstein B, Bigner DD (2009). IDH1 and IDH2 mutations in gliomas. N. Engl. J. Med..

[5] Marcucci G, Maharry K, Wu YZ, Radmacher MD, Mrozek K, Margeson D, Holland KB, Whitman SP, Becker H, Schwind S, Metzeler KH, Powell BL, Carter TH, Kolitz JE, Wetzler M, Carroll AJ, Baer MR, Caligiuri MA, Larson RA, Bloomfield CD (2010). IDH1 and IDH2 gene mutations identify novel molecular subsets within de novo cytogenetically normal acute myeloid leukemia: A Cancer and Leukemia Group B study. J. Clin. Oncol..

[6] Borger DR, Tanabe KK, Fan KC, Lopez HU, Fantin VR, Straley KS, Schenkein DP, Hezel AF, Ancukiewicz M, Liebman HM, Kwak EL, Clark JW, Ryan DP, Deshpande V, Dias-Santagata D, Ellisen LW, Zhu AX, Iafrate AJ (2012). Frequent mutation of isocitrate dehydrogenase (IDH)1 and IDH2 in cholangiocarcinoma identified through broad-based tumor genotyping. Oncologist.

[7] Wang P, Dong Q, Zhang C, Kuan PF, Liu Y, Jeck WR, Andersen JB, Jiang W, Savich GL, Tan TX, Auman JT, Hoskins JM, Misher AD, Moser CD, Yourstone SM, Kim JW, Cibulskis K, Getz G, Hunt HV, Thorgeirsson SS, Roberts LR, Ye D, Guan KL, Xiong Y, Qin LX, Chiang DY (2013). Mutations in isocitrate dehydrogenase 1 and 2 occur frequently in intrahepatic cholangiocarcinomas and share hypermethylation targets with glioblastomas. Oncogene.

[8] Xu W, Yang H, Liu Y, Yang Y, Wang P, Kim SH, Ito S, Yang C, Wang P, Xiao MT, Liu LX, Jiang WQ, Liu J, Zhang JY, Wang B, Frye S, Zhang Y, Xu YH, Lei QY, Guan KL, Zhao SM, Xiong Y (2011). Oncometabolite 2-hydroxyglutarate is a competitive inhibitor of alpha-ketoglutarate-dependent dioxygenases. Cancer Cell.

[9] Figueroa ME, Abdel-Wahab O, Lu C, Ward PS, Patel J, Shih A, Li Y, Bhagwat N, Vasanthakumar A, Fernandez HF, Tallman MS, Sun Z, Wolniak K, Peeters JK, Liu W, Choe SE, Fantin VR, Paietta E, Lowenberg B, Licht JD, Godley LA, Delwel R, Valk PJ, Thompson CB, Levine RL, Melnick A (2010). Leukemic IDH1 and IDH2 mutations result in a hypermethylation phenotype, disrupt TET2 function, and impair hematopoietic differentiation. Cancer Cell.

[10] Jiao Y, Pawlik TM, Anders RA, Selaru FM, Streppel MM, Lucas DJ, Niknafs N, Guthrie VB, Maitra A, Argani P, Offerhaus G, Roa JC, Roberts LR, Gores GJ, Popescu I, Alexandrescu ST, Dima S, Fassan M, Simbolo M, Mafficini A, Capelli P, Lawlor RT, Ruzzenente A, Guglielmi A, Tortora G, de Braud F, Scarpa A, Jarnagin W, Klimstra D, Karchin R, Velculescu VE, Hruban RH, Vogelstein B, Kinzler KW, Papadopoulos N, Wood LD (2013). Exome sequencing identifies frequent inactivating mutations in BAP1, ARID1A and PBRM1 in intrahepatic cholangiocarcinomas. Nat. Genet..

[11] Rizzo A, Ricci AD, Brandi G (2020). Futibatinib, an investigational agent for the treatment of intrahepatic cholangiocarcinoma: Evidence to date and future perspectives. Expert Opin. Investig. Drugs.

[12] Massa A, Varamo C, Vita F, Tavolari S, Peraldo-Neia C, Brandi G, Rizzo A, Cavalloni G, Aglietta M (2020). Evolution of the experimental models of cholangiocarcinoma. Cancers (Basel).

[13] Kassner A, Thornhill RE (2010). Texture analysis: A review of neurologic MR imaging applications. AJNR Am. J. Neuroradiol..

[14] Chae HD, Park CM, Park SJ, Lee SM, Kim KG, Goo JM (2014). Computerized texture analysis of persistent part-solid ground-glass nodules: Differentiation of preinvasive lesions from invasive pulmonary adenocarcinomas. Radiology.

[15] Weiss GJ, Ganeshan B, Miles KA, Campbell DH, Cheung PY, Frank S, Korn RL (2014). Noninvasive image texture analysis differentiates K-ras mutation from pan-wildtype NSCLC and is prognostic. PLoS ONE.

[16] Ranjbar S, Ning S, Zwart CM, Wood CP, Weindling SM, Wu T, Mitchell JR, Li J, Hoxworth JM (2018). Computed tomography-based texture analysis to determine human papillomavirus status of oropharyngeal squamous cell carcinoma. J. Comput. Assist. Tomogr..

[17] Miles KA, Ganeshan B, Rodriguez-Justo M, Goh VJ, Ziauddin Z, Engledow A, Meagher M, Endozo R, Taylor SA, Halligan S, Ell PJ, Groves AM (2014). Multifunctional imaging signature for V-KI-RAS2 Kirsten rat sarcoma viral oncogene homolog (KRAS) mutations in colorectal cancer. J. Nucl. Med..

[18] Ozkan E, West A, Dedelow JA, Chu BF, Zhao W, Yildiz VO, Otterson GA, Shilo K, Ghosh S, King M, White RD, Erdal BS (2015). CT gray-level texture analysis as a quantitative imaging biomarker of epidermal growth factor receptor mutation status in adenocarcinoma of the lung. AJR Am. J. Roentgenol..

[19] Sacconi B, Anzidei M, Leonardi A, Boni F, Saba L, Scipione R, Anile M, Rengo M, Longo F, Bezzi M, Venuta F, Napoli A, Laghi A, Catalano C (2017). Analysis of CT features and quantitative texture analysis in patients with lung adenocarcinoma: A correlation with EGFR mutations and survival rates. Clin. Radiol..

[20] Ng F, Ganeshan B, Kozarski R, Miles KA, Goh V (2013). Assessment of primary colorectal cancer heterogeneity by using whole-tumor texture analysis: contrast-enhanced CT texture as a biomarker of 5-year survival. Radiology.

[21] Lee SJ, Zea R, Kim DH, Lubner MG, Deming DA, Pickhardt PJ (2018). CT texture features of liver parenchyma for predicting development of metastatic disease and overall survival in patients with colorectal cancer. Eur. Radiol..

[22] Jakola AS, Zhang YH, Skjulsvik AJ, Solheim O, Bo HK, Berntsen EM, Reinertsen I, Gulati S, Forander P, Brismar TB (2018). Quantitative texture analysis in the prediction of IDH status in low-grade gliomas. Clin. Neurol. Neurosurg..

[23] Yu J, Shi Z, Lian Y, Li Z, Liu T, Gao Y, Wang Y, Chen L, Mao Y (2017). Noninvasive IDH1 mutation estimation based on a quantitative radiomics approach for grade II glioma. Eur. Radiol..

[24] Zhu Y (2018). Predicting idh mutation status of intrahepatic cholangiocarcinomas based on contrast-enhanced CT features. Eur. Radiol..

[25] Kim SA, Lee JM, Lee KB, Kim SH, Yoon SH, Han JK, Choi BI (2011). Intrahepatic mass-forming cholangiocarcinomas: Enhancement patterns at multiphasic CT, with special emphasis on arterial enhancement pattern–correlation with clinicopathologic findings. Radiology.

[26] Ros PR, Buck JL, Goodman ZD, Ros AM, Olmsted WW (1988). Intrahepatic cholangiocarcinoma: Radiologic––pathologic correlation. Radiology.

[27] Fujita N, Asayama Y, Nishie A, Ishigami K, Ushijima Y, Takayama Y, Okamoto D, Moirta K, Shirabe K, Aishima S, Wang H, Oda Y, Honda H (2017). Mass-forming intrahepatic cholangiocarcinoma: Enhancement patterns in the arterial phase of dynamic hepatic CT—Correlation with clinicopathological findings. Eur. Radiol..

[28] Barry B, Buch K, Soto JA, Jara H, Nakhmani A, Anderson SW (2014). Quantifying liver fibrosis through the application of texture analysis to diffusion weighted imaging. Magn. Reson. Imaging.

[29] Yu H, Touret AS, Li B, O'Brien M, Qureshi MM, Soto JA, Jara H, Anderson SW (2017). Application of texture analysis on parametric T1 and T2 maps for detection of hepatic fibrosis. J. Magn. Reson. Imaging.

[30] Witten, I. & Frank, E. *Data mining: practical machine learning tools and techniques. Morgan Kaugmann Series in Data Management Systems* 2nd edn, 150–151 (Elsevier, 2005).

[31] Ganeshan B, Burnand K, Young R, Chatwin C, Miles K (2011). Dynamic contrast-enhanced texture analysis of the liver: Initial assessment in colorectal cancer. Invest. Radiol..

[32] Simpson AL, Adams LB, Allen PJ, D'Angelica MI, DeMatteo RP, Fong Y, Kingham TP, Leung U, Miga MI, Parada EP, Jarnagin WR, Do RK (2015). Texture analysis of preoperative CT images for prediction of postoperative hepatic insufficiency: A preliminary study. J. Am. Coll. Surg..

[33] Daginawala N, Li B, Buch K, Yu H, Tischler B, Qureshi MM, Soto JA, Anderson S (2016). Using texture analyses of contrast enhanced CT to assess hepatic fibrosis. Eur. J. Radiol..

[34] Ganeshan B, Miles KA (2013). Quantifying tumour heterogeneity with CT. Cancer Imaging.

[35] Ng F, Kozarski R, Ganeshan B, Goh V (2013). Assessment of tumor heterogeneity by CT texture analysis: Can the largest cross-sectional area be used as an alternative to whole tumor analysis?. Eur. J. Radiol..

[36] Ganeshan B, Miles KA, Young RC, Chatwin CR (2007). In search of biologic correlates for liver texture on portal-phase CT. Acad. Radiol..

[37] Ganeshan B, Miles KA, Young RC, Chatwin CR (2009). Texture analysis in non-contrast enhanced CT: Impact of malignancy on texture in apparently disease-free areas of the liver. Eur. J. Radiol..

[38] Lubner MG, Smith AD, Sandrasegaran K, Sahani DV, Pickhardt PJ (2017). CT texture analysis: Definitions, applications, biologic correlates, and challenges. Radiographics.

[39] Gevaert O, Xu J, Hoang CD, Leung AN, Xu Y, Quon A, Rubin DL, Napel S, Plevritis SK (2012). Non-small cell lung cancer: Identifying prognostic imaging biomarkers by leveraging public gene expression microarray data–methods and preliminary results. Radiology.

[40] Liu Y, Kim J, Balagurunathan Y, Li Q, Garcia AL, Stringfield O, Ye Z, Gillies RJ (2016). Radiomic features are associated with EGFR mutation status in lung adenocarcinomas. Clin. Lung Cancer.

[41] Haralick R, Shanmugam K (1973). Textural features for image classification. IEEE Trans. Syst. Man Cybern..

[42] Noble WS (2006). What is a support vector machine?. Nat. Biotechnol..

[43] Li Z, Wang Y, Yu J, Guo Y, Cao W (2017). Deep Learning based Radiomics (DLR) and its usage in noninvasive IDH1 prediction for low grade glioma. Sci. Rep..

[44] Ahn SJ, Kim JH, Park SJ, Han JK (2016). Prediction of the therapeutic response after FOLFOX and FOLFIRI treatment for patients with liver metastasis from colorectal cancer using computerized CT texture analysis. Eur. J. Radiol..

